# POSITIVE AND NEGATIVE SOCIAL INTERACTIONS ON MENTAL HEALTH IN OLDER KOREAN AMERICANS: GENDER DIFFERENCES

**DOI:** 10.1093/geroni/igz038.1900

**Published:** 2019-11-08

**Authors:** Nan Sook Park, Yuri Jang, Min-Kyoung Rhee, David Chiriboga, Soondool Chung

**Affiliations:** 1 University of South Florida, Tampa, Florida, United States; 2 University of Southern California, Los Angeles, California, United States; 3 Ewha Womans University, Seoul, Korea, Republic of

## Abstract

While there is substantial documentation of a positive relationship between objective social engagement and mental health, relatively little is known about how perceived quality of social interactions affects mental health and how men and women differ. Considering the gap, the purpose of this study was to investigate gender difference in how social interactions associate with self-rated mental health in older Korean Americans. Data came from a survey with older Korean Americans aged 60 or over that included 713 men and 1437 women living in five sites (California, New York, Texas, Hawaii, and Florida), conducted during 2017−2018. In multiple regression models run separately for men and women, self-rated mental health on a five-point scale (excellent/very good/good/fair/poor) was regressed on four blocks of variables: socio-demographic characteristics (age, marital status, education, financial status, self-rated health, and region), immigration-related variables (length of stay in the U.S. and acculturation), social engagement (family network, friend network, and activity participation), and perceived quality of social interactions (positive or negative family interactions and negative community interactions). In the final models with all covariates, younger age, more years in education, better physical health, higher levels of acculturation, and more positive family interactions were commonly associated with more positively rated mental health for both men and women. For women, stronger family network and fewer negative family interactions were additional contributors. Results suggest that negative and positive indicators of family interactions differentially affect self-rated mental health for older Korean American men and women.

